# Providing the prediction of traffic influence on children’s behavioral abnormalities 

**Published:** 2019-08

**Authors:** Maede Musazade Gasgari

**Affiliations:** ^*a*^Faculty of Transportation, Azad University of Shahriyar, Iran.

## Abstract

**Background:**

Traffic pollution is one of the integral part in today s civilized life, which can be referred as air or noise pollution, and also psychosocial and social contamination. Attentively to various contaminated areas, it can be concluded that the significant harm to traffic-based pollution is for children and should be carefully monitored for child injuries. One of the aspects of children s psychosocial and social harm from today s life is the abnormalities and behavioral disorders existing among them. In this research, we tried to investigate the behavioral disorders of children exposed to traffic injuries which cause on behavioral abnormalities and created in children. So some strategies to prevent harm to children should be shown in this research.

**Methods:**

For this purpose, a descriptive-correlation questionnaire was developed and correlations were made. From1430 schoolchildren in the 10th, 17th and 2nd schools of Tehran area as the most infected and most polluted areas in Tehran, 305 of them were evaluated.

**Results:**

The result of this measure showed that there is a significant relationship between traffic pollution such as air pollution, noise pollution, other contaminations and behavioral abnormalities in children. And as every child has more time in traffic and the level of exposure to it is more, the nervous behavior will be weaker. For example, the results of the u Mann-Whitney test on the comparison of the average of the ratings of the two groups of children who were more than thirty minutes and less than thirty minutes exposed to traffic pollution showed that the average rank of the second group is 118.61, and the average rating of the first group is 187.09.

**Conclusions:**

The results showed that in children who have more than thirty minutes expose to the traffic, there seen more behavioral abnormalities.

**Keywords:**

Traffic influence, Children’s behavioral abnormalities, Traffic pollution

